# Validity of a Smartphone Application in Calculating Measures of Heart Rate Variability

**DOI:** 10.3390/s22249883

**Published:** 2022-12-15

**Authors:** Andreas T. Himariotis, Kyle F. Coffey, Sabrina E. Noel, David J. Cornell

**Affiliations:** 1 Health Assessment Laboratory, University of Massachusetts Lowell, Lowell, MA 01854, USA; 2 Department of Physical Therapy and Kinesiology, University of Massachusetts Lowell, Lowell, MA 01854, USA; 3 Department of Biomedical and Nutritional Sciences, University of Massachusetts Lowell, Lowell, MA 01854, USA

**Keywords:** heart rate monitor, autonomic nervous system function, R–R interval data, artifact corrections

## Abstract

The purpose of the current study was to determine the concurrent validity of the Elite HRV smartphone application when calculating heart rate variability (HRV) metrics in reference to an independent software criterion. A total of 5 minutes of R–R interval and natural log of root mean square of the successive differences (lnRMSSD) resting HRV data were simultaneously collected using two Polar H10 heart rate monitors (HRMs) in both the seated and supine positions from 22 participants (14 males, 8 females). One H10 HRM was paired with a Polar V800 watch and one with the Elite HRV application. When no artifact correction was applied, significant, but *small*, differences in the lnRMSSD data were observed between the software in the seated position (*p* = 0.022), and *trivial* and nonstatistically significant differences were observed in the supine position (*p* = 0.087). However, significant differences (*p* > 0.05) in the lnRMSSD data were no longer identifiable in either the seated or the supine positions when applying Very Low, Low, or Automatic artifact-correction filters. Additionally, *excellent* agreements (ICC_3,1_ = 0.938 − 0.998) and *very strong* to *near-perfect* (*r* = 0.889 − 0.997) relationships were observed throughout all correction levels. The Elite HRV smartphone application is a valid tool for calculating resting lnRMSSD HRV metrics.

## 1. Introduction

Heart rate variability (HRV) is a metric derived from the variability of R–R intervals from an electrocardiogram (ECG). HRV metrics can be divided into time-domain and frequency-domain measures [1], but overall, they are utilized as a noninvasive method for assessing autonomic nervous system (ANS) function [2]. The use of various HRV metrics has grown tremendously in recent years as a result of advances in noninvasive physiological monitors capable of collecting R–R intervals without the use of electrocardiography devices [3,4]. In addition, recent research has suggested that time-domain measures of HRV are capable of producing valid and reliable HRV metrics utilizing relatively short samples (e.g., <5 min) [5]. Specifically, the time-domain HRV metric of the root mean square of successive differences between normal heartbeats (RMSSD) [1] has been shown to be stable [5], reliable [6], and accurate [7] during spontaneous breathing patterns [8]. As such, the RMSSD metric of HRV has been utilized by sports scientists to track workload and recovery and to modify the training of athletes [9,10,11,12,13,14], as well as by researchers to prescribe exercise and to track improvements in health and fitness [9,15].

Up until recently, the processing of raw R–R interval data to calculate HRV metrics still required separate software packages and the need to download and/or transfer data for processing, creating a barrier to the widespread utilization of HRV among practitioners. However, the creation of smartphone applications that can pair with physiological monitors via Bluetooth technology has allowed for the processing of R–R interval data and the calculation of HRV metrics in real-time [16,17,18,19,20,21]. These smartphone applications have now given sports scientists tools that allow for the daily monitoring of HRV metrics on an individual level [22]. In spite of this, there is a lack of validation of the processing techniques and calculations within the smartphone applications themselves in the scientific literature. Specifically, without standardizing the hardware that is collecting the raw R–R interval data and then comparing how the smartphone application processes the R–R interval data and calculates the HRV metrics, the smartphone applications themselves have yet to be truly validated. Since these smartphone applications utilize proprietary algorithms to conduct these processing techniques and calculations, it is important to ensure that these smartphone applications are operating similarly to established software packages, such as Kubios HRV software [23]. Furthermore, it is also unclear how artifacts in the R–R interval data are corrected within these smartphone applications, which has been known to influence the calculation of various HRV metrics [24].

Therefore, the objective of this project was to determine the concurrent validity of the Elite HRV smartphone application (Elite HRV Inc., Asheville, NC, USA), which has grown in popularity in the scientific literature [17,18,19,20,21,25], in reference to a criterion based on Kubios HRV software (Kubios, Ltd., Kuopio, Finland) [23]. Specifically, the primary purpose of the current study was to examine the concurrent validity of the Elite HRV smartphone application when processing and calculating HRV metrics. A secondary purpose was to determine whether the artifact-correction level as applied to the R–R interval data influences the concurrent validity of the Elite HRV smartphone application when processing and calculating HRV metrics.

## 2. Materials and Methods

### 2.1. Participants

A total of 24 participants (15 males, 9 females) between the ages of 18 to 36 years old were recruited from the university campus community as volunteers to participate in this study (age: 22.38 ± 3.54 years; height: 172.21 ± 8.26 cm; body mass: 77.53 ± 17.22 kg). Eligibility criteria included anyone who was at least 18 years of age and willing to provide informed consent. Any individuals who were previously diagnosed with a cardiovascular disease or cardiac arrhythmia and/or were taking any medications influencing the cardiovascular system were excluded from this study. All study protocols for this study were approved by the Institutional Review Board (IRB) at the University of Massachusetts Lowell, and all participants provided written informed consent before data collection. This study was conducted in accordance with the Declaration of Helsinki. All participants were given opportunities to ask questions regarding the study before any data were collected.

### 2.2. Experimental Protocol

Prior to the collection of HRV measurements, anthropometric data were first collected from each participant. After collecting these descriptive data, participants then began the experimental protocol of collecting 5 min of R–R interval data during resting conditions, first in the supine, and then in the seated, position. In order to be consistent with field-based HRV data collection methods [12,17,18], the breathing rate of each participant was not controlled (i.e., spontaneous breathing patterns). All R–R interval data were collected utilizing two Polar H10 HRMs (Polar Electro, Kempele, Finland). However, one H10 HRM was paired via Bluetooth to a Polar V800 watch (Polar Electro, Kempele, Finland), and one H10 HRM was paired via Bluetooth to the Elite HRV application (Elite HRV, Ashville, NC, USA) on a Google Pixel 4a smartphone (Google, Mountain View, CA, USA). Participants were asked to abstain from any vigorous physical activity for 48 h before their testing sessions.

### 2.3. Procedures

#### 2.3.1. Anthropometric Data Collection

Anthropometric descriptive data were collected according to guidelines from the American College of Sports Medicine (ACSM) [26]. Specifically, body mass (kg) and height (cm) data were collected using a Detecto scale and stadiometer (Detecto, Webb City, MO, USA) to the nearest 0.1 kg and 0.5 cm, respectively.

#### 2.3.2. R–R Data Collection

**Figure 1:**
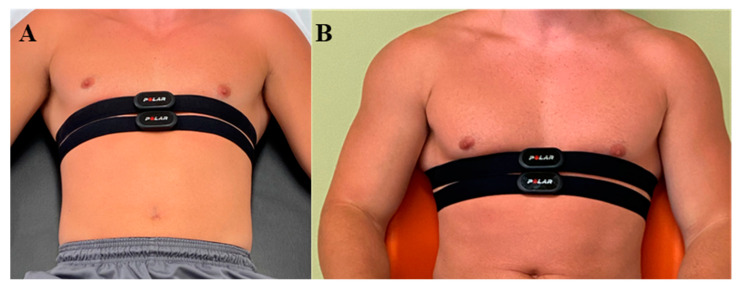
Configuration of Polar H10 heart rate monitors in the supine (**A**) and seated (**B**) positions.

Two separate Polar H10 HRMs were placed over the xiphoid process of each participant superiorly and inferiorly to each other, and R–R interval data were simultaneously collected in both the supine (Figure 1A) and seated (Figure 1B) positions according to recommendations in the literature [27]. Specifically, after a five-minute stabilization period, 5 min of R–R interval data were collected in each position with the participant quietly resting. Controls for breathing rate were not performed by the researchers in the current study, as our procedure was consistent with field-based approaches to collecting HRV data [12,17,18].

#### 2.3.3. HRV Processing

Heart rate (HR) and HRV metrics derived from the R–R interval data collected by the Polar H10 HRM linked to the Elite HRV smartphone application (Elite HRV Inc., Asheville, NC, USA) were processed entirely within the smartphone application. However, HRV metrics derived from the R–R interval data collected by the Polar H10 HRM linked to the Polar V800 watch were downloaded to an external laptop and processed via Kubios HRV 3.5.0 software (Kubios, Ltd., Kuopio, Finland).

In addition, HRV metrics were derived utilizing multiple processing threshold filters within Kubios HRV 3.5.0 software [23], which compares every R–R interval value to the median of the R–R interval time series. If a specific R–R interval value differed from the median by more than a specified threshold value, that interval was then corrected. These R–R interval processing filters included the following thresholds: No, Very Low (0.45 s), Low (0.35 s), and Automatic. Multiple R–R interval processing threshold filters were utilized in order to determine whether the artifact-correction level of R–R interval data processing explained potential discrepancies in the criterion validity of the HRV metrics processed within the Elite HRV smartphone application.

Since breathing rate was not controlled in the current study, the root mean square of differences between successive heartbeats (RMSSD) was the time-domain HRV parameter utilized [1] as previous research has demonstrated the RMSSD parameter to be the best for short-term HRV samples because it is more stable, [5] reliable, [6] and accurate [7] during spontaneous breathing patterns.

### 2.4. Statistical Analyses

Examination of the histograms and Q–Q plots indicated that the HRV metrics of RMSSD violated the assumption normality. Therefore, the natural log (ln) of RMSSD (lnRMSSD) was calculated and utilized instead, which normalized the distribution [28] and is consistent with the scientific literature [10]. HR and R–R interval data did not violate normality assumptions.

To determine the concurrent validity of the HRV metrics (HR, R–R intervals, and lnRMSSD) derived by the Elite HRV smartphone application in reference to Kubios HRV software criterion, a variety of statistical approaches were utilized, as recommended in the scientific literature [29]. These statistical approaches were utilized for measurements collected in both the supine and seated positions. In addition, these approaches were repeated at each level of artifact-correction filtering (No, Very Low, Low, Automatic) to determine whether the concurrent validity of the Elite HRV smartphone application changed based on how the R–R interval data was processed. 

First, paired *t*-tests examined absolute agreement [29] and Hedges’ *g* effect sizes determined the magnitude of potential differences [30] in the HRV metrics derived from the two software platforms. Additionally, bivariate Pearson correlations (*r*) and coefficients of determination (*R^2^*) were utilized to determine the level of association and variance shared between the HRV metrics derived by these platforms [31]. Relative levels of agreement of the HRV metrics derived from the two software platforms were examined by utilizing two-way mixed model [32] intraclass correlation coefficients (ICC_3,1_) [31] and by constructing Bland–Altman plots [33] using the mean bias and 95% limits of agreement (LoA) [34]. Lastly, percentage errors (PEs) were calculated to express the mean bias as a percentage of the mean measure of the two methods [35]:

(1) $$PE=\left(\frac{100\times \left[1.96\times SD\;of\;Bias\right]}{\left[\frac{\left(Mean\;Elite\;HRV+Mean\;Kubios\;HRV\right)}{2}\right]}\right)$$

An alpha of 0.05 was used to determine statistically significant differences in the HRV metrics derived from the two software platforms, and Hedges’ *g* effect sizes were interpreted as: *very large*: *g* ≥ 2.0; *large*: 2.0 > *g* ≥ 1.2; *moderate*: 1.2 > *g* ≥ 0.6; *small*: 0.6 > *g* ≥ 0.2; or *trivial*: *g* < 0.2 [36]. Correlation coefficients (*r*) were interpreted as: *near-perfect*: *r* ≥ 0.9; *very strong*: 0.9 < *r* ≥ 0.70; *strong*: 0.70 > *r* ≥ 0.50; *moderate*: 0.50 > *r* ≥ 0.30; *small*: 0.30 > *r* ≥ 0.10; or *trivial*: *r* < 0.10 [37]. ICCs were interpreted as: *excellent*: ICC ≥ 0.90; *good*: 0.90 > ICC ≥ 0.75; *moderate*: 0.75 > ICC ≥ 0.50; *fair*: 0.50 > ICC ≥ 0.40; or *poor*: ICC < 0.40 [38]. Finally, a clinically acceptable level of PEs was determined using a threshold of <30% [35]. All statistical analyses were conducted using Microsoft Excel (Microsoft Corp., Redmond, WA, USA) and SPSS v28 statistical software (IBM Corp., Armonk, NY, USA).

## 3. Results

Based on the previously defined exclusion criteria, one male participant was excluded from the study due to a cardiac arrythmia observed during data collection. In addition, one female participant was identified as an outlier [39] and was subsequently removed from the statistical analyses. Finally, due to device malfunction during data collection, one female participant was excluded from the statistical analyses of the data collected in the seated position only.

### 3.1. Supine–No Correction

#### 3.1.1. HR Data

**Figure 2:**
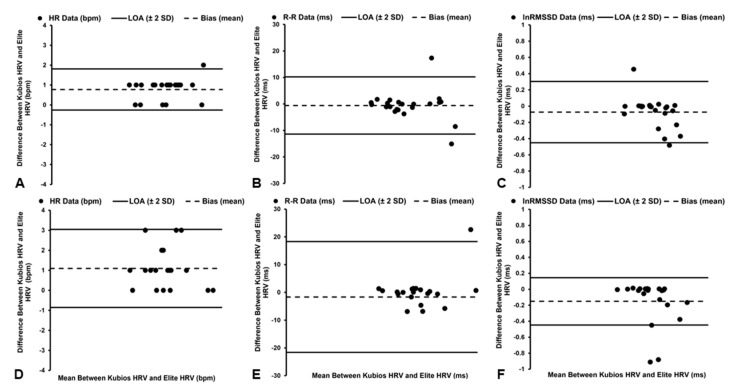
Bland–Altman plots demonstrating the mean bias and limits of agreement (LoA) of heart rate (HR, **A**), R–R interval (**B**), and natural log of root mean square of successive differences (lnRMSSD, **C**) data in the supine position and the mean bias and limits of agreement (LoA) of HR (**D**), R–R interval (**E**), and lnRMSSD (**F**) data in seated position when utilizing No artifact correction.

Statistically significant (*t*_21_ = 6.859, *p* < 0.001), but *trivial*, differences in the HR data were observed between the software platforms (Table 1), with the Elite HRV smartphone application demonstrating a mean bias of +0.78 bpm and 1.60% PEs (Figure 2A). However, *excellent* agreement (ICC_3,1_ = 1.000, [95% CI: 0.999, 1.000]) and a *near-perfect* relationship were identified between the software platforms (*r* = 0.999, *p* < 0.001), with the Elite HRV smartphone application accounting for 99% of the variance (*R*^2^ = 0.99) in the HR data derived by Kubios HRV software.

#### 3.1.2. R–R Interval Data

*Trivial* and nonsignificant (*t*_21_ = 0.494, *p* = 0.627) differences in R–R interval data were observed between the software platforms (Table 1), with a mean bias of −0.58 ms and 1.11% PEs (Figure 2B). *Excellent* agreement (ICC_3,1_ = 1.000, [95% CI: 1.000, 1.000]) and a *near-perfect* relationship were also identified between the software platforms (*r* = 1.000, *p* < 0.001), with the Elite HRV smartphone application accounting for 100% of the variance (*R*^2^ = 1.00) in R–R interval data derived by Kubios HRV software.

#### 3.1.3. lnRMSSD Data

*Trivial* and nonsignificant (*t*_21_ = 1.798, *p* = 0.087) differences in lnRMSSD data were observed between the software platforms (Table 1), with the Elite HRV smartphone application demonstrating a mean bias of −0.07 ms and 8.95% PEs (Figure 2C). In addition, *excellent* agreement (ICC_3,1_ = 0.970, [95% CI: 0.928, 0.988]) and a *near-perfect* relationship were identified between the software packages (*r* = 0.957, *p* < 0.001), with the Elite HRV smartphone application accounting for 92% of the variance (*R*^2^ = 0.92) in lnRMSSD data derived by Kubios HRV software.

### 3.2. Seated–No Correction

#### 3.2.1. HR Data

Although a significant a difference (*t*_20_ = 5.043, *p* < 0.001) in HR data was observed between the software platforms, along with a mean bias of +1.10 bpm and 2.87% PEs (Figure 2D), these differences were considered *trivial* (Table 1). In addition, *excellent* agreement (ICC_3,1_ = 0.998, [95% CI: 0.996, 0.999]) and a *near-perfect* relationship were identified between the software platforms (*r* = 0.997, *p* < 0.001), with the Elite HRV smartphone application accounting for 99% of the variance (*R*^2^ = 0.99) in HR data derived by Kubios HRV software.

#### 3.2.2. R–R Interval Data

*Trivial* and nonsignificant (*t*_20_ = 0.745, *p* = 0.465) differences in R–R interval data were observed between the software platforms (Table 1), with the Elite HRV smartphone application demonstrating a mean bias of −1.66 ms and 2.18% PEs (Figure 2E). In addition, *excellent* agreement (ICC_3,1_ = 0.999, [95% CI: 0.998, 1.000]) and a *near-perfect* relationship were identified between the software platforms (*r* = 0.998, *p* < 0.001), with the Elite HRV smartphone application accounting for 99% of the variance (*R*^2^ = 0.99) in R–R interval data derived by Kubios HRV software.

#### 3.2.3. lnRMSSD Data

*Small* and statistically significant (*t*_20_ = 2.483, *p* = 0.022) differences in lnRMSSD data were observed between the software platforms (Table 1), with the Elite HRV smartphone application demonstrating a mean bias of −0.15 ms and 13.28% PEs (Figure 2F). However, *excellent* agreement (ICC_3,1_ = 0.938, [95% CI: 0.846, 0.975]) and a *near-perfect* relationship were identified between the software platforms (*r* = 0.889, *p* < 0.001), with the Elite HRV smartphone application accounting for 79% of the variance (*R*^2^ = 0.79) in lnRMSSD data derived by Kubios HRV software.

### 3.3. Supine–Very Low Correction Level

#### 3.3.1. HR Data

**Figure 3:**
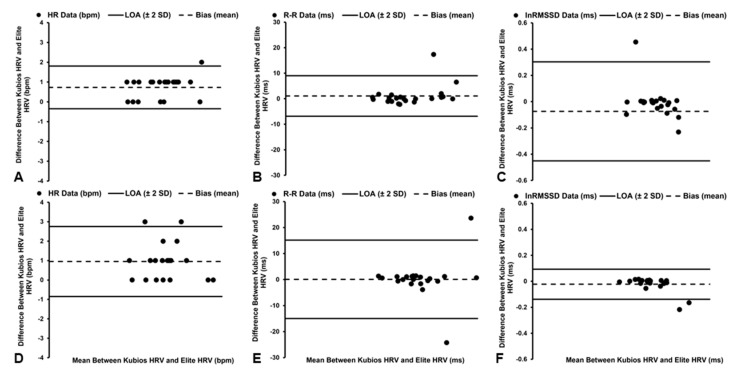
Bland–Altman plots demonstrating the mean bias and limits of agreement (LoA) of heart rate (HR, **A**), R–R interval (**B**), and natural log of root mean square of successive differences (lnRMSSD, **C**) data in the supine position and the mean bias and limits of agreement (LoA) of HR (**D**), R–R interval (**E**), and lnRMSSD (**F**) data in seated position when utilizing Very Low artifact-correction level.

Significant (*t*_21_ = 6.197, *p* < 0.001), but *trivial*, differences were observed in HR data between the software platforms (Table 2), with the Elite HRV smartphone application demonstrating a mean bias of +0.73 bpm and 1.67% PEs (Figure 3A). However, *excellent* agreement (ICC_3,1_ = 1.000, [95% CI: 0.999, 1.000]) and a *near-perfect* relationship were identified between the software platforms (*r* = 0.999, *p* < 0.001), with the Elite HRV smartphone application accounting for 99% of the variance (*R*^2^ = 0.99) in HR data derived by Kubios HRV software.

#### 3.3.2. R–R Interval Data

*Trivial* and nonsignificant (*t*_21_ = 1.226, *p* = 0.234) differences were observed in R–R interval data between the software platforms (Table 2), with a mean bias of −1.06 ms and 0.81% PEs (Figure 3B). In addition, *excellent* agreement (ICC_3,1_ = 1.000, [95% CI: 1.000, 1.000]) and a *near-perfect* relationship were identified between the software packages (*r* = 1.000, *p* < 0.001), with the Elite HRV smartphone application accounting for 100% of the variance (*R*^2^ = 1.00) in R–R interval data derived by Kubios HRV software.

#### 3.3.3. lnRMSSD Data

*Trivial* and nonsignificant (*t*_21_ = 0.402, *p* = 0.692) differences in lnRMSSD data were observed between the software platforms (Table 2), with the Elite HRV smartphone application demonstrating a mean bias of −0.01 ms and 5.59% PEs (Figure 3C). *Excellent* agreement (ICC_3,1_ = 0.988, [95% CI: 0.970, 0.995]) and a *near-perfect* relationship were identified between the software packages (*r* = 0.980, *p* < 0.001), with the Elite HRV smartphone application accounting for 96% of the variance (*R*^2^ = 0.96) in lnRMSSD data derived by Kubios HRV software.

### 3.4. Seated—Very Low Correction Level

#### 3.4.1. HR Data

Significant (*t*_20_ = 4.740, *p* < 0.001), but *trivial*, differences were observed in HR data between the software platforms (Table 2), with the Elite HRV smartphone application demonstrating a mean bias of +0.95 bpm and a 2.65% PEs (Figure 3D). However, *excellent* agreement (ICC_3,1_ = 0.999, [95% CI: 0.997, 0.999]) and a *near-perfect* relationship were identified between the software packages (*r* = 0.997, *p* < 0.001), with the Elite HRV smartphone application accounting for 99% of the variance (*R*^2^ = 0.99) in HR data derived by Kubios HRV software.

#### 3.4.2. R–R Interval Data

*Trivial* and nonsignificant (*t*_20_ = 0.063, *p* = 0.951) differences were observed in R–R interval data between the software platforms (Table 2), with a mean bias of +0.10 ms and 1.65% PEs (Figure 3E). In addition, *excellent* agreement (ICC_3,1_ = 0.999, [95% CI: 0.999, 1.000]) and a *near-perfect* relationship were identified between the software packages (*r* = 0.999, *p* < 0.001), with the Elite HRV smartphone application accounting for 99% of the variance (*R*^2^ = 0.99) in R–R interval data derived by Kubios HRV software.

#### 3.4.3. lnRMSSD Data

*Trivial* and nonsignificant (*t*_20_ = 1.748, *p* = 0.096) differences in lnRMSSD data were observed between the software platforms (Table 2), with the Elite HRV smartphone application demonstrating a mean bias of −0.02 ms and 2.86% PEs (Figure 3F). In addition, *excellent* agreement (ICC_3,1_ = 0.997, [95% CI: 0.993, 0.999]) and a *near-perfect* relationship were identified between the software platforms (*r* = 0.997, *p* < 0.001), with the Elite HRV smartphone application accounting for 99% of the variance (*R*^2^ = 0.99) in lnRMSSD data derived by Kubios HRV software.

### 3.5. Supine—Low Correction Level

#### 3.5.1. HR Data

**Figure 4:**
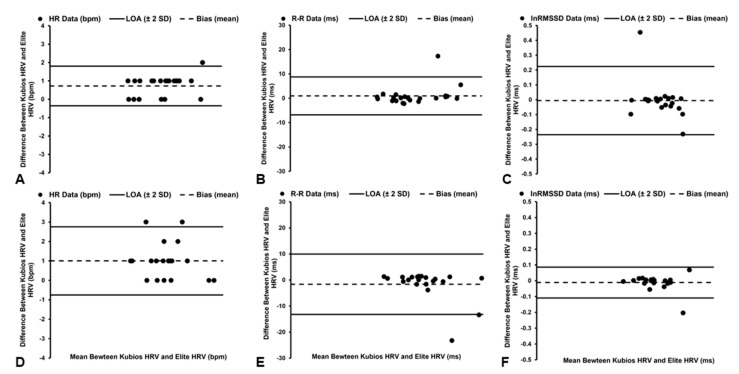
Bland–Altman plots demonstrating the mean bias and limits of agreement (LoA) of heart rate (HR, **A**), R–R interval (**B**), and natural log of root mean square of successive differences (lnRMSSD, **C**) data in the supine position and the mean bias and limits of agreement (LoA) of HR (**D**), R–R interval (**E**), and lnRMSSD (**F**) data in seated position when utilizing Low artifact-correction level.

Significant (*t*_21_ = 6.197, *p* < 0.001), but *trivial*, differences were observed in HR data between the software platforms (Table 3), with the Elite HRV smartphone application demonstrating a mean bias of +0.73 bpm and 1.67% PEs (Figure 4A). In addition, *excellent* agreement (ICC_3,1_ = 1.000, [95% CI: 0.999, 1.000]) and a *near-perfect* relationship were identified between the software packages (*r* = 0.999, *p* < 0.001), with the Elite HRV smartphone application accounting for 99% of the variance (*R*^2^ = 0.99) in HR data derived by Kubios HRV software.

#### 3.5.2. R–R Interval Data

*Trivial* and nonsignificant (*t*_21_ = 1.139, *p* = 0.268) differences were observed in R–R interval data between the software platforms (Table 3), with a mean bias of +0.97 ms and 0.80% PEs (Figure 4B). Additionally, *excellent* agreement (ICC_3,1_ = 1.000, [95% CI: 1.000, 1.000]) and a *near-perfect* relationship were identified between the software packages (*r* = 1.000, *p* < 0.001), with the Elite HRV smartphone application accounting for 100% of the variance (*R*^2^ = 1.00) in R–R interval data derived by Kubios HRV software.

#### 3.5.3. lnRMSSD Data

*Trivial* and nonsignificant (*t*_21_ = 0.244, *p* = 0.810) differences in lnRMSSD data were observed between the software platforms (Table 3), with the Elite HRV smartphone application demonstrating a mean bias of −0.01 ms and 5.50% PEs (Figure 4C). *Excellent* agreement (ICC_3,1_ = 0.988, [95% CI: 0.971, 0.995]) and a *near-perfect* relationship were identified between the software packages (*r* = 0.979, *p* < 0.001), with the Elite HRV smartphone application accounting for 96% of the variance (*R*^2^ = 0.96) in lnRMSSD data derived by Kubios HRV software.

### 3.6. Seated—Low Correction Level

#### 3.6.1. HR Data

Significant (*t*_20_ = 5.123, *p* < 0.001), but *trivial*, differences in HR data were observed between the software platforms (Table 3), with a mean bias of +1.00 bpm and 2.58% PEs (Figure 4D). However, *excellent* agreement (ICC_3,1_ = 0.999, [95% CI: 0.997, 0.999]) and a *near-perfect* relationship were identified between the software packages (*r* = 0.997, *p* < 0.001), with the Elite HRV smartphone application accounting for 99% of the variance (*R*^2^ = 0.99) in HR data derived by Kubios HRV software.

#### 3.6.2. R–R Interval Data

*Trivial* and nonsignificant (*t*_20_ = 1.248, *p* = 0.226) differences were observed in R–R interval data between the software platforms (Table 3), with the Elite HRV smartphone application demonstrating a mean bias of −1.61 ms and 1.27% PEs (Figure 4E). *Excellent* agreement (ICC_3,1_ = 1.000, [95% CI: 0.999, 1.000]) and a *near-perfect* relationship were identified between the software packages (*r* = 1.000, *p* < 0.001), with the Elite HRV smartphone application accounting for 100% of the variance (*R*^2^ = 1.00) in R–R interval data derived by Kubios HRV software.

#### 3.6.3. lnRMSSD Data

*Trivial* and nonsignificant (*t*_20_ = 1.010, *p* = 0.324) differences were observed in lnRMSSD data between the software platforms (Table 3), with the Elite HRV smartphone application demonstrating a mean bias of −0.01 ms and 2.41% PEs (Figure 4F). In addition, *excellent* agreement (ICC_3,1_ = 0.998, [95% CI: 0.995, 0.999]) and a *near-perfect* relationship were identified between the software packages (*r* = 0.996, *p* < 0.001), with the Elite HRV smartphone application accounting for 99% of the variance (*R*^2^ = 0.99) in lnRMSSD data derived by Kubios HRV software.

### 3.7. Supine—Automatic Correction

#### 3.7.1. HR Data

**Figure 5:**
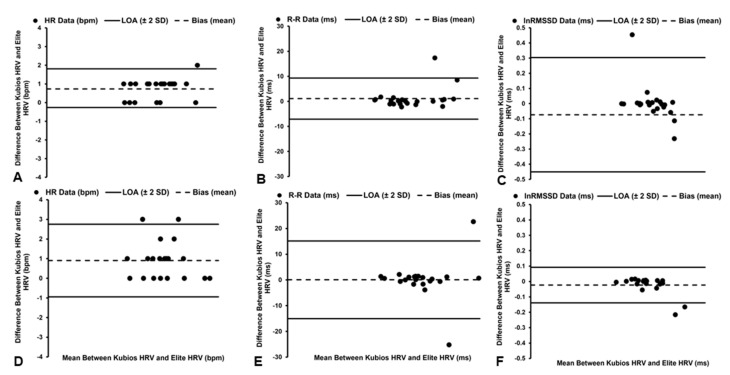
Bland–Altman plots demonstrating the mean bias and limits of agreement (LoA) of heart rate (HR, **A**), R–R interval (**B**), and natural log of root mean square of successive differences (lnRMSSD, **C**) data in the supine position and the mean bias and limits of agreement (LoA) of HR (**D**), R–R interval (**E**), and lnRMSSD (**F**) data in seated position when utilizing Automatic artifact correction.

Significant (*t*_21_ = 6.197, *p* < 0.001), but *trivial*, differences were observed in HR data between the software platforms (Table 4), with the Elite HRV smartphone application demonstrating a mean bias of +0.73 bpm and 1.67% PEs (Figure 5A). Additionally, *excellent* agreement (ICC_3,1_ = 1.000, [95% CI: 0.999, 1.000]) and a *near-perfect* relationship were identified between the software packages (*r* = 0.999, *p* < 0.001), with the Elite HRV smartphone application accounting for 99% of the variance (*R*^2^ = 0.99) in HR data derived by Kubios HRV software.

#### 3.7.2. R–R Interval Data

*Trivial* and nonsignificant (*t*_21_ = 1.232, *p* = 0.231) differences were observed in R–R interval data between the software platforms (Table 4), with a mean bias of +1.10 ms and 0.84% PEs (Figure 5B). *Excellent* agreement (ICC_3,1_ = 1.000, [95% CI: 1.000, 1.000]) and a *near-perfect* relationship were identified between the software platforms (*r* = 1.000, *p* < 0.001), with the Elite HRV smartphone application accounting for 100% of the variance (*R*^2^ = 1.00) in R–R interval data derived by Kubios HRV software.

#### 3.7.3. lnRMSSD Data

*Trivial* and nonsignificant (*t*_21_ = 0.080, *p* = 0.937) differences were observed in lnRMSSD data between the software platforms (Table 4), with the Elite HRV smartphone application demonstrating a mean bias of −0.002 ms and 5.49% PEs (Figure 5C). *Excellent* agreement (ICC_3,1_ = 0.988, [95% CI: 0.971, 0.995]) and a *near-perfect* relationship were identified between the software platforms (*r* = 0.982, *p* < 0.001), with the Elite HRV smartphone application accounting for 96% of the variance (*R*^2^ = 0.96) in lnRMSSD data derived by Kubios HRV software.

### 3.8. Seated—Automatic Correction

#### 3.8.1. HR Data

Significant (*t*_20_ = 4.394, *p* < 0.001), but *trivial*, differences in HR data were observed between the software platforms (Table 4), with the Elite HRV smartphone application demonstrating a mean bias of +0.91 bpm and 2.72% PEs (Figure 5D). In addition, *excellent* agreement (ICC_3,1_ = 0.999, [95% CI: 0.996, 0.999]) and a *near-perfect* relationship were identified between the software platforms (*r* = 0.997, *p* < 0.001), with the Elite HRV smartphone application accounting for 99% of the variance (*R*^2^ = 0.99) in HR data derived by Kubios HRV software.

#### 3.8.2. R–R Interval Data

*Trivial* and nonsignificant (*t*_20_ = 0.034, *p* = 0.973) differences in R–R interval data were observed between the software platforms (Table 4), with a mean bias of +0.06 ms and 1.65% PEs (Figure 5E). Additionally, *excellent* agreement (ICC_3,1_ = 0.999, [95% CI: 0.999, 1.000]) and a *near-perfect* relationship were identified between the software platforms (*r* = 0.999, *p* < 0.001), with the Elite HRV smartphone application accounting for 99% of the variance (*R*^2^ = 0.99) in R–R interval data derived by Kubios HRV software.

#### 3.8.3. lnRMSSD Data

*Trivial* and nonsignificant (*t*_20_ = 1.767, *p* = 0.093) differences in lnRMSSD data were observed between the software platforms (Table 4), with the Elite HRV smartphone application demonstrating a mean bias of −0.02 ms and 2.85% PEs (Figure 5F). However, *excellent* agreement (ICC_3,1_ = 0.997, [95% CI: 0.993, 0.999]) and a *near-perfect* relationship were identified between the software platforms (*r* = 0.997, *p* < 0.001), with the Elite HRV smartphone application accounting for 99% of the variance (*R*^2^ = 0.99) in lnRMSSD data derived by Kubios HRV software.

## 4. Discussion

Given the rise of various smartphone technologies that collect various health and fitness measures [40] and the increased number of noninvasive physiological monitors capable of collecting valid R–R interval data without the use of electrocardiography devices [3,4], there has been a dramatic increase in the utilization of HRV measures by researchers [3]. This increased presence in the scientific literature has been further accompanied by smartphone applications that can instantaneously derive HRV measures, eliminating potential barriers in HRV utilization for practitioners [3]. Given the proprietary nature of the processes utilized in these applications, it is important to ensure that the algorithms used in these smartphone applications to process these R–R interval data and calculate HRV metrics are accurate and comparable to previously established methods. However, examination of the validity of the smartphone applications themselves has yet to be conducted in the scientific literature. Therefore, the primary purpose of the current study was to examine the concurrent validity of the Elite HRV smartphone application when processing and calculating HRV metrics with reference to third-party Kubios HRV software as the criterion [23].

*Excellent* agreement was identified between the Elite HRV smartphone application and Kubios HRV software when examining HR, R–R interval, and lnRMSSD data collected in both supine and seated positions in the current study, suggesting adequate relative validity of the Elite HRV smartphone application. However, differing results were observed in regard to absolute validity. Specifically, significant differences in HR data were identified between the software platforms in both the supine and seated positions although these differences were *trivial* in magnitude. Furthermore, although no differences in R–R interval data were identified between the software platforms in both the supine and seated positions, which is consistent with previous research [18], *small* differences in lnRMSSD data were identified between the software platforms, with these differences being statistically significant in the seated position. Therefore, although the PEs remained below the clinically acceptable level (PEs < 30%) [35], questionable concurrent absolute validity of the Elite HRV smartphone application was apparent when comparing the calculated HRV metrics to noncorrected data.

Guzik et al. [19] identified similar results, with significant differences observed in R–R interval and RMSSD data derived by the Elite HRV smartphone application and external HRV software. These differences remained apparent after applying correction filters to remove ectopic beats, which is recommended when processing R–R interval data and calculating HRV metrics [27], leading the authors to suggest that the Elite HRV smartphone application is not a valid method of processing R–R interval data and calculating HRV metrics [19]. However, these results differed from those of both Gambassi et al. [18] and Perotta et al. [17], which demonstrated adequate concurrent validity of the Elite HRV smartphone application when comparing the RMSSD data derived by the Elite HRV smartphone application to RMSSD data derived by Kubios HRV software as the criterion. In addition, in contrast to Guzik et al. [19], Perotta et al. [17] also utilized a Moderate artifact-correction filter when processing the R–R interval data before deriving HRV metrics, suggesting that this discrepancy in the scientific literature, and the questionable concurrent validity of the Elite HRV smartphone application in the current study, could simply be due to differences in processing techniques between researchers.

That said, it should also be noted that all of these studies [17,18,19] compared R–R interval data collected by a HRM that was paired to the Elite HRV smartphone application to R–R interval data collected using an electrocardiography device and processed via Kubios HRV software. As such, given the utilization of differing devices to collect the raw R–R interval data, none of these previous studies actually determined the concurrent validity of the Elite HRV smartphone application itself. Since this smartphone application utilizes proprietary algorithms to process these R–R interval data and calculate HRV metrics, it is important to not only determine the concurrent validity of this application, but also to understand how this application processes R–R interval data and corrects potential ectopic beats.

Consequently, a secondary purpose of the current study was to determine whether the artifact-correction level applied to the R–R interval data influences the concurrent validity of the Elite HRV smartphone application when processing and calculating HRV metrics. After applying a Very Low artifact-correction filter to the R–R interval data in Kubios HRV software [23], *near-perfect* relationships and *excellent* agreement were observed between the Elite HRV smartphone application and Kubios HRV software when examining HR, R–R interval, and lnRMSSD data collected in both supine and seated positions. In addition, *trivial* and nonsignificant differences in R–R interval and lnRMSSD data were observed between the software platforms, and although significant differences in HR data remained apparent, the magnitude of these differences were *trivial*. Similar results were identified when applying a Low or Automatic artifact-correction filter to the R–R interval data in Kubios HRV software [23] as well. Given the fact that utilizing an artifact filter to correct ectopic beats is recommended when processing R–R interval data and calculating HRV metrics [27], these results indicate that utilizing the Elite HRV smartphone application leads to the same HRV metrics as Kubios HRV software when applying any level of artifact correction. 

Therefore, the results of the current study suggest that the Elite HRV smartphone application holds both relative and absolute concurrent validity when calculating HRV metrics. These results hold notable practical relevance for strength and conditioning professionals and other health and fitness practitioners wishing to collect measures of HRV in a field-based manner. Specifically, these results suggest that sports scientists could utilize the Elite HRV smartphone application when tracking the workload and recovery of athletes, and that clinicians and practitioners could utilize the Elite HRV smartphone application to prescribe exercise and track improvements in health and fitness. In addition, these results indicate that the Elite HRV smartphone application could be utilized by researchers hoping to collect measures of HRV in a nonlaboratory setting.

Several limitations of the current study should be noted. First, the sample population was limited to participants between the ages of 19–26 years old who were recruited from a university campus. Therefore, future research should confirm concurrent validity of the Elite HRV smartphone application when processing R–R interval data and calculating HRV metrics collected from differently aged populations. Additionally, previous studies have investigated the concurrent validity of various devices when collecting R–R interval data in other testing positions, such as standing [41] or tilted [42]. Previous studies have also examined other time-domain HRV metrics, such as the standard deviation of the interbeat interval of normal sinus beats (SDNN) [18,19], or even frequency-domain measures of HRV [18]. As such, future research should examine the concurrent validity of the Elite HRV smartphone application when processing R–R interval data collected in different positions and when calculating other relevant HRV metrics. However, it should also be noted that the supine and seated positions were chosen in the current study for ecological reasons, as R–R interval data are most frequently collected in these positions under spontaneous breathing patterns [9,10,11,12,13,14], and the RMSSD metric has shown to be stable [5], reliable [6], and accurate [7] during spontaneous breathing patterns [8]. Finally, although measures of HRV are assessed in nonexercising conditions [27], assessing HRV during postexercise recovery situations is also common [6,43,44]. Thus, future research should also examine the concurrent validity of the Elite HRV smartphone application when processing R–R interval data (and calculating HRV metrics) collected during postexercise recovery conditions.

## 5. Conclusions

In conclusion, although researchers in the sports science domain have been utilizing measures of HRV to track the workload and recovery of athletes [9,10,11,12,13,14], as well as to prescribe exercise programming and to track improvements in health and fitness [9,15], the feasibility for practitioners to utilize measures of HRV has been limited by barriers in the processing of raw R–R interval data and the calculation of HRV metrics. Due to the ability of the Elite HRV smartphone application to instantaneously process R–R interval data and calculate HRV metrics, the concurrent validity demonstrated in this study suggests that this smartphone application platform could be utilized among researchers and practitioners alike as a more feasible, convenient, inexpensive, and easy-to-use method of examining the ANS function of individuals.

## Figures and Tables

**Table 1:** No Correction—Descriptive data, bias, error, and agreement between software packages.

**Supine (*n* = 22)**							
	**Kubios HRV** **(mean ± SD)**	**Elite HRV** **(mean ± SD)**	**Effect Size ^§^** **(Hedges’ *g*)**	**Bias** **(mean ± SD)**	**95% LoA**	**PEs** **(%)**	**Agreement ^‡^** **(ICC_3,1_)**
HR (bpm)	64.18 ± 13.14	64.95 ± 13.29 ^†^	0.06 *trivial*	+0.78 ± 0.53	−0.26, 1.81	1.60	1.000 *excellent*
R–R (ms)	973.82 ± 203.87	973.24 ± 202.93	<0.01 *trivial*	−0.58 ± 5.52	−11.39, 10.23	1.11	1.000 *excellent*
lnRMSSD (ms)	4.25 ± 0.61	4.17 ± 0.51	0.14 *trivial*	−0.07 ± 0.19	−0.45, 0.30	8.95	0.970 *excellent*
**Seated (*n* = 21)**							
	**Kubios HRV** **(mean ± SD)**	**Elite HRV** **(mean ± SD)**	**Effect Size ^§^** **(Hedges’ *g*)**	**Bias** **(mean ± SD)**	**95% LoA**	**PEs** **(%)**	**Agreement ^‡^** **(ICC_3,1_)**
HR (bpm)	67.48 ± 12.41	68.57 ± 12.30 ^†^	0.09 *trivial*	+1.10 ± 1.00	−0.86, 3.05	2.87	0.998 *excellent*
R–R (ms)	915.57 ± 169.19	913.91 ± 169.16	0.01 *trivial*	−1.66 ± 10.20	−21.64, 18.33	2.18	0.999 *excellent*
lnRMSSD (ms)	4.19 ± 0.61	4.04 ± 0.54 ^†^	0.26 *small*	−0.15 ± 0.28	−0.70, 0.40	13.28	0.938 *excellent*

Note: 95% LoA, 95% limits of agreement; PEs, percentage errors; ICC_3,1_, two-way mixed model intraclass correlation coefficient; HR, heart rate; R–R, R–R intervals; lnRMSSD, natural log of root mean square of successive differences. ^†^ Indicates significant difference between the software platforms (*p* < 0.05). ^‡^ ICC interpretation based on the guidelines from Koo et al. [38] ^§^ Hedges’ *g* interpretation based on the guidelines from Hopkins et al. [36].

**Table 2:** Very Low Correction Level—Descriptive data, bias, error, and agreement between software packages.

**Supine (*n* = 22)**							
	**Kubios HRV** **(Mean ± SD)**	**Elite HRV** **(Mean ± SD)**	**Effect Size ^§^** **(Hedges’ *g*)**	**Bias** **(Mean ± SD)**	**95% LoA**	**PEs** **(%)**	**Agreement ^‡^** **(ICC_3,1_)**
HR (bpm)	64.23 ± 13.08	64.95 ± 13.29 ^†^	0.05 *trivial*	+0.73 ± 0.55	−0.35, 1.81	1.67	1.000 *excellent*
R–R (ms)	972.18 ± 201.47	973.24 ± 202.93	0.01 *trivial*	+1.06 ± 4.04	−6.86, 8.97	0.81	1.000 *excellent*
lnRMSSD (ms)	4.18 ± 0.56	4.17 ± 0.51	0.02 *trivial*	−0.01 ± 0.12	−0.24, 0.22	5.59	0.988 *excellent*
Corrected Artifacts (no.)	0.27 ± 0.63	0.41 ± 1.92					
**Seated (*n* = 21)**							
	**Kubios HRV** **(mean ± SD)**	**Elite HRV** **(mean ± SD)**	**Effect Size ^§^** **(Hedges’ *g*)**	**Bias** **(mean ± SD)**	**95% LoA**	**PEs** **(%)**	**Agreement ^‡^** **(ICC_3,1_)**
HR (bpm)	67.62 ± 12.37	68.57 ± 12.30 ^†^	0.08 *trivial*	+0.95 ± 0.92	−0.85, 2.76	2.65	0.999 *excellent*
R–R (ms)	913.81 ± 168.18	913.91 ± 169.16	<0.01 *trivial*	+0.10 ± 7.68	−14.95, 15.16	1.65	0.999 *excellent*
lnRMSSD (ms)	4.06 ± 0.58	4.04 ± 0.54	0.04 *trivial*	−0.02 ± 0.06	−0.14, 0.09	2.86	0.997 *excellent*
Corrected Artifacts (no.)	0.76 ± 1.61	0.05 ± 0.22					

Note: 95% LoA, 95% limits of agreement; PEs, percentage errors; ICC_3,1_, two-way mixed model intraclass correlation coefficient; HR, heart rate; RR, R–R intervals; lnRMSSD, natural log of root mean square of successive differences. ^†^ Indicates significant difference between the software platforms (*p* < 0.05). ^‡^ ICC interpretation based on the guidelines from Koo et al. [38] ^§^ Hedges’ *g* interpretation based on the guidelines from Hopkins et al. [36].

**Table 3:** Low Correction Level—Descriptive data, bias, error, and agreement between software packages.

**Supine (*n* = 22)**							
	**Kubios HRV** **(mean ± SD)**	**Elite HRV** **(mean ± SD)**	**Effect Size ^§^** **(Hedges’ *g*)**	**Bias** **(mean ± SD)**	**95% LoA**	**PEs** **(%)**	**Agreement ^‡^** **(ICC_3,1_)**
HR (bpm)	64.23 ± 13.08	64.95 ± 13.29 ^†^	0.05 *trivial*	+0.73 ± 0.55	−0.35, 1.81	1.67	1.000 *excellent*
R–R (ms)	972.27 ± 201.61	973.24 ± 202.93	0.01 *trivial*	+0.97 ± 3.98	−6.83, 8.76	0.80	1.000 *excellent*
lnRMSSD (ms)	4.18 ± 0.56	4.17 ± 0.51	0.01 *trivial*	−0.01 ± 0.12	−0.24, 0.22	5.50	0.988 *excellent*
Corrected Artifacts (no.)	0.41 ± 0.80	0.41 ± 1.92					
**Seated (*n* = 21)**							
	**Kubios HRV** **(mean ± SD)**	**Elite HRV** **(mean ± SD)**	**Effect Size ^§^** **(Hedges’ *g*)**	**Bias** **(mean ± SD)**	**95% LoA**	**PEs** **(%)**	**Agreement ^‡^** **(ICC_3,1_)**
HR (bpm)	67.57 ± 12.45	68.57 ± 12.30 ^†^	0.08 *trivial*	+1.00 ± 0.89	−0.75, 2.75	2.58	0.999 *excellent*
R–R (ms)	915.52 ± 171.91	913.91 ± 169.16	0.01 *trivial*	−1.61 ± 5.91	−13.19, 9.97	1.27	1.000 *excellent*
lnRMSSD (ms)	4.05 ± 0.55	4.04 ± 0.54	0.02 *trivial*	−0.01 ± 0.05	−0.11, 0.09	2.41	0.998 *excellent*
Corrected Artifacts (no.)	2.62 ± 8.27	0.05 ± 0.22					

Note: 95% LoA, 95% limits of agreement; PEs, percentage errors; ICC_3,1_, two-way mixed model intraclass correlation coefficient; HR, heart rate; R–R, R–R intervals; lnRMSSD, natural log of root mean square of successive differences. ^†^ Indicates significant difference between the software platforms (*p* < 0.05). ^‡^ ICC interpretation based on the guidelines from Koo et al. [38] ^§^ Hedges’ *g* interpretation based on the guidelines from Hopkins et al. [36].

**Table 4:** Automatic Correction—Descriptive data, bias, error, and agreement between software packages.

**Supine (*n* = 22)**							
	**Kubios HRV** **(mean ± SD)**	**Elite HRV** **(mean ± SD)**	**Effect Size ^§^** **(Hedges’ *g*)**	**Bias** **(mean ± SD)**	**95% LoA**	**PEs** **(%)**	**Agreement ^‡^** **(ICC_3,1_)**
HR (bpm)	64.23 ± 13.08	64.95 ± 13.29 ^†^	0.05 *trivial*	+0.73 ± 0.55	−0.35, 1.81	1.67	1.000 *excellent*
R–R (ms)	972.14 ± 201.54	973.24 ± 202.93	0.01 *trivial*	+1.10 ± 4.19	−7.11, 9.32	0.84	1.000 *excellent*
lnRMSSD (ms)	4.17 ± 0.57	4.17 ± 0.51	<0.01 *trivial*	−0.002 ± 0.12	−0.23, 0.23	5.49	0.988 *excellent*
Corrected Artifacts (no.)	0.68 ± 1.13	0.41 ± 1.92					
**Seated (*n* = 21)**							
	**Kubios HRV** **(mean ± SD)**	**Elite HRV** **(mean ± SD)**	**Effect Size ^§^** **(Hedges’ *g*)**	**Bias** **(mean ± SD)**	**95% LoA**	**PEs** **(%)**	**Agreement ^‡^** **(ICC_3,1_)**
HR (bpm)	67.67 ± 12.42	68.57 ± 12.30 ^†^	0.07 *trivial*	+0.91 ± 0.94	−0.94, 2.75	2.72	0.999 *excellent*
R–R (ms)	913.86 ± 168.38	913.91 ± 169.16	<0.01 *trivial*	+0.06 ± 7.70	−15.04, 15.15	1.65	0.999 *excellent*
lnRMSSD (ms)	4.06 ± 0.58	4.04 ± 0.54	0.04 *trivial*	−0.02 ± 0.06	−0.14, 0.09	2.85	0.997 *excellent*
Corrected Artifacts (no.)	0.29 ± 0.72	0.05 ± 0.22					

Note: 95% LoA, 95% limits of agreement; PEs, percentage errors; ICC_3,1_, two-way mixed model intraclass correlation coefficient; HR, heart rate; R–R, R–R intervals; lnRMSSD, natural log of root mean square of successive differences. ^†^ Indicates significant difference between the software platforms (*p* < 0.05). ^‡^ ICC interpretation based on the guidelines from Koo et al. [38] ^§^ Hedges’ *g* interpretation based on the guidelines from Hopkins et al. [36].

## Data Availability

The data presented in this study are available upon request from the corresponding author.
